# Perifoveal interdigitation zone loss in hydroxychloroquine toxicity leads to subclinical bull’s eye lesion appearance on near-infrared reflectance imaging

**DOI:** 10.1007/s10633-017-9615-9

**Published:** 2017-11-09

**Authors:** Avenell L. Chew, Danuta M. Sampson, Enid Chelva, Jane C. Khan, Fred K. Chen

**Affiliations:** 1 Centre for Ophthalmology and Visual Science (Incorporating Lions Eye Institute), The University of Western Australia, Crawley, WA 6009 Australia; 2 Department of Medical Technology and Physics, Sir Charles Gairdner Hospital, Nedlands, WA 6009 Australia; 3 Department of Ophthalmology, Royal Perth Hospital, Perth, WA 6000 Australia

**Keywords:** Fundus autofluorescence, Bull’s eye maculopathy, Adaptive optics, *En face* optical coherence tomography, Multifocal electroretinography, Microperimetry

## Abstract

**Purpose:**

To characterize the ultrastructural and functional correlates of hydroxychloroquine (HCQ)-induced subclinical bull’s eye lesion seen on near-infrared reflectance (NIR) imaging.

**Methods:**

An asymptomatic 54-year-old male taking HCQ presented with paracentral ring-like scotoma, abnormal multifocal electroretinography (mfERG) and preserved ellipsoid zone on optical coherence tomography (OCT). Dense raster OCT was performed to create *en face* reflectivity maps of the interdigitation zone. Macular Integrity Assessment (MAIA) microperimetry and mfERG findings were compared with NIR imaging, *en face* OCT, retinal thickness profiles and wave-guiding cone density maps derived from flood-illumination adaptive optics (AO) retinal photography.

**Results:**

The bull’s eye lesion is an oval annular zone of increased reflectivity on NIR with an outer diameter of 1450 µm. This region corresponds exactly to an area of preserved interdigitation zone reflectivity in *en face* OCT images and of normal cone density on AO imaging. Immediately surrounding the bull’s eye lesion is an annular zone (3°–12° eccentricity) of depressed retinal sensitivity on MAIA and reduced amplitude density on mfERG. Wave-guiding cone density at 2° temporal was 25,400 per mm^2^. This declined rapidly to 12,900 and 1200 per mm^2^ at 3° and 4°.

**Conclusion:**

Multimodal imaging illustrated pathology in the area surrounding the NIR bull’s eye, characterized by reduced reflectance, wave-guiding cone density and retinal function. Further studies are required to investigate whether the bull’s eye on NIR imaging and *en face* OCT is prominent or consistent enough for diagnostic use.

**Electronic supplementary material:**

The online version of this article (doi:10.1007/s10633-017-9615-9) contains supplementary material, which is available to authorized users.

## Introduction

The triad of ring scotoma, bull’s eye maculopathy (BEM) on fundoscopy and the loss of the ellipsoid zone on optical coherence tomography (OCT) are recognized features of hydroxychloroquine (HCQ) toxicity. However, the diagnosis of toxicity can be uncertain in a subset of patients with ring-like scotoma without BEM or ellipsoid zone loss on OCT scan.

Wong et al. [1] described an imaging sign of HCQ toxicity characterized by a subclinical bull’s eye lesion only visible on near-infrared reflectance (NIR) imaging in patients with ring-like scotoma, no BEM on fundus examination and only mild ellipsoid zone changes on OCT scan. Although the anatomical and functional correlates of the classic BEM in HCQ toxicity have been well described [2], the ultrastructural basis and functional consequences of the NIR bull’s eye lesion have not been investigated in detail. Wong et al. [1] observed that the boundary of the NIR bull’s eye lesion did not co-localize with ellipsoid zone changes on OCT. They concluded that biochemical changes related to early HCQ toxicity may be accountable for the increased reflectance [1]. However, the relationships between the NIR bull’s eye lesion, interdigitation zone (cone tips) integrity and retinal function have not yet been investigated to exclude other possible explanations of this sign.

Herein, we use multimodal imaging to examine the structure–function correlation in a patient with NIR bull’s eye lesion due to early HCQ toxicity. Our hypothesis is that NIR bull’s eye lesion arises from changes within the interdigitation zone. We have therefore chosen a case of early HCQ toxicity with ring-like scotoma, but preserved ellipsoid zone to test our hypothesis.

## Methods

### Patient recruitment

A 54-year-old Caucasian male of European descent with systemic lupus erythematosus presented with suspected HCQ toxicity in May 2014. He underwent complete ophthalmic examination and multimodal imaging.

All procedures were performed in accordance with the tenets of the Declaration of Helsinki, and informed consent was obtained from the study participants described in this report. Control sample data were obtained from prospective multimodal imaging studies approved by the University of Western Australia Human Research Ethics Office (Trial Number: RA/4/1/7226, RA/4/1/5455, RA/4/1/7457) and the Sir Charles Gairdner Human Research Ethics Committee (Trial Number: 1998:115).

### Functional assessment

Best-corrected visual acuity was measured using the Early Treatment of Diabetic Retinopathy Study (ETDRS) chart (Lighthouse International, New York, USA). Automated visual fields were performed in a darkened room using the Humphrey Field Analyzer (HFA II 750, Carl Zeiss Meditec GmbH, Germany). The central 10–2 threshold test grid was used, and the Swedish Interactive Threshold Algorithm (SITA) standard was employed to measure sensitivity to white targets of Goldmann size III.

Fundus-controlled microperimetry was performed using a scanning laser ophthalmoscope, CenterVue MAIA (CenterVue, Padova, Italy), in a dedicated darkened psychophysics room. The fixation target was a 2° red circle broken into four segments; the dim white background had a luminance level of 1.27 cd/m^2^, but the range of Δ*L* (luminance difference between stimulus and background) was 0.08–317.5 cd/m^2^ producing a dynamic range of 0–36 dB. Stimulus size was Goldmann III; duration was 200 ms; and testing protocol was a 4–2 staircase threshold strategy. The test grid used in microperimetry is identical to the Humphrey central 10–2 threshold test grid consisting of 68 loci arranged in a Cartesian pattern projected onto the central 20° of the macula.

Multifocal electroretinography (mfERG) was performed using the VERIS Science V6.3.2 system (Electro Diagnostic Imaging Inc., San Mateo, CA, USA) with the patient fully dilated. The test stimulus used consisted of an array of 103 retinal-scaled hexagons covering 45° of visual angle. Burian–Allen corneal contact lens electrodes (Hansen Ophthalmic Development Lab, Coralville, IA, USA) were used to record visually evoked responses incorporating the guidelines set out by the 2011 International Society for Clinical Electrophysiology of Vision standard for clinical mfERG [3]. The 103 local mfERG responses were grouped into six concentric rings of equal eccentricity centered on the fovea. These rings are defined as follows: ring 1 (a 3° wide central hexagon), 0°–1.5° eccentricity; ring 2, 1.5°–4° eccentricity; ring 3, 4°–8° eccentricity; ring 4, 8°–12° eccentricity; ring 5, 12°–17° eccentricity; and ring 6, 17°–22° eccentricity.

### Structural assessment

Near-infrared reflectance (NIR), short-wavelength autofluorescence (SWAF) and spectral domain optical coherence tomography (OCT) were acquired in high-resolution mode using the Spectralis HRA + OCT (Heidelberg Engineering, Heidelberg, Germany).

*En face* minimum projection intensity reflectivity maps of the ellipsoid and interdigitation zones within the foveal region were created from OCT B-scans using the HEYEX 3D viewing module in Spectralis (version 6.0.9.0). The layer of interest is manually selected on a single B-scan to generate an intensity profile of that layer within the region of the macula imaged.

Wave-guiding cone outer segment tips were visualized using a flood-illumination adaptive optics (AO) fundus camera (rtx1, Imagine Eye, Orsay, France). Overlapping 4° × 4° averaged single AO frame was taken at 2° of gaze separation to cover the central 12° of visual angle. Each single AO frame is also converted to a 4° × 4° color map showing cone density distribution (AODetect, Imagine Eye). All single AO frames from the same eye were aligned, merged and stitched to create a wide-field AO montage using the MosaicJ [4] plugin of ImageJ (Laboratory for Optical and Computational Instrumentation, San Jose, USA). The relative coordinates of these frames were used to stitch single color maps to create a wide-field color map of cone density. Both the wide-field AO montage and wide-field color maps were overlaid on the NIR image from the Spectralis HRA + OCT using vessel landmarks for registration. This alignment allowed precise localization of regions of interest on the wide-field AO montages to enable cone density measurement at precisely measured visual angles from the anatomical foveal center rather than gross estimation of retinal location relative to the preferred retinal locus based on gaze direction alone.

Axial lengths were measured using the IOLMaster 500 (Carl Zeiss Meditec GmbH, Germany). These measurements were used to define pixel:µm ratio which is required for generating a density map from the onboard AODetect software (Imagine Eye, Orsay, France). Cone outer segment signals within the wide-field AO montage were identified manually for cone density measurement within a sampling window of 50 × 50 µm, chosen at 1° intervals from the foveal center in the horizontal and vertical meridians. The outer segments of cones from foveal center to 1° eccentricity (1 µm in size) are too small to be resolved by the rtx1 camera since the resolution is only 3 µm.

### Structure–function analysis

All measurements of lateral dimensions and localization of region of interest were in degrees of visual angle. Linear dimensions were converted to visual angle based on the conversion factor of 300 µm on the retina = 1° visual angle.

All measurements derived from the patient were compared to control sample statistics as described by Crawford and Howell [5]. Modified *t* tests were performed to test the hypothesis that the measurement values from this patient came from a control population [6]. A *p* value of < 0.05 is set for one-tailed distribution given a priori assumption of a decline in measured value due to HCQ toxicity.

The control sample for retinal sensitivity (MAIA) consists of 36 healthy individuals with a mean age of 53 (standard deviation, SD = 16, 20 males, 17 females) years. The control sample for amplitude density (mfERG) consists of right and left eyes of 13 healthy individuals with a mean age of 50 (SD = 5, 10 males, 3 females) years. The control sample for retinal thickness (OCT) consists of 28 healthy males with a mean age of 48 (SD = 18) years. The control sample for cone density (AO imaging) consists of 24 healthy individuals with a mean age of 55 (SD = 15.8, 14 males, 10 females).

## Results

The patient presented for routine screening of HCQ toxicity in 2014. He had no visual symptoms after continuous HCQ dosing for 12.4 years at a daily dose of 4.3 mg/kg of total body weight and a cumulative dose of 1808 g. He had no other significant medical comorbidities such as renal or liver disease and no history of uveitis or episcleritis. He had no family history of retinal disease. Best-corrected visual acuity was 85 letters (20/20) bilaterally. Dilated fundus examination revealed no clinical evidence of maculopathy, previous inflammatory eye disease or lupus chorioretinopathy. Axial length of the right eye was 24.15 mm in the affected patient.

## Functional assessment

The 10–2 Humphrey visual field test demonstrated an incomplete paracentral ring-like scotoma in each eye. Increased thresholds were detected at 3°, 5°, 7° and 9° eccentricities. Mean deviations were −2.73 dB (*p* < 0.02) and −3.04 dB (*p* < 0.02) in right and left eyes, respectively. Of 68 test loci, 22 (32%) had elevated thresholds on pattern deviation plot (top 5 percentile) in each eye. There were more relative scotomas in the nasal than in the temporal visual field (Supp Fig 1).

**Fig. 1:**
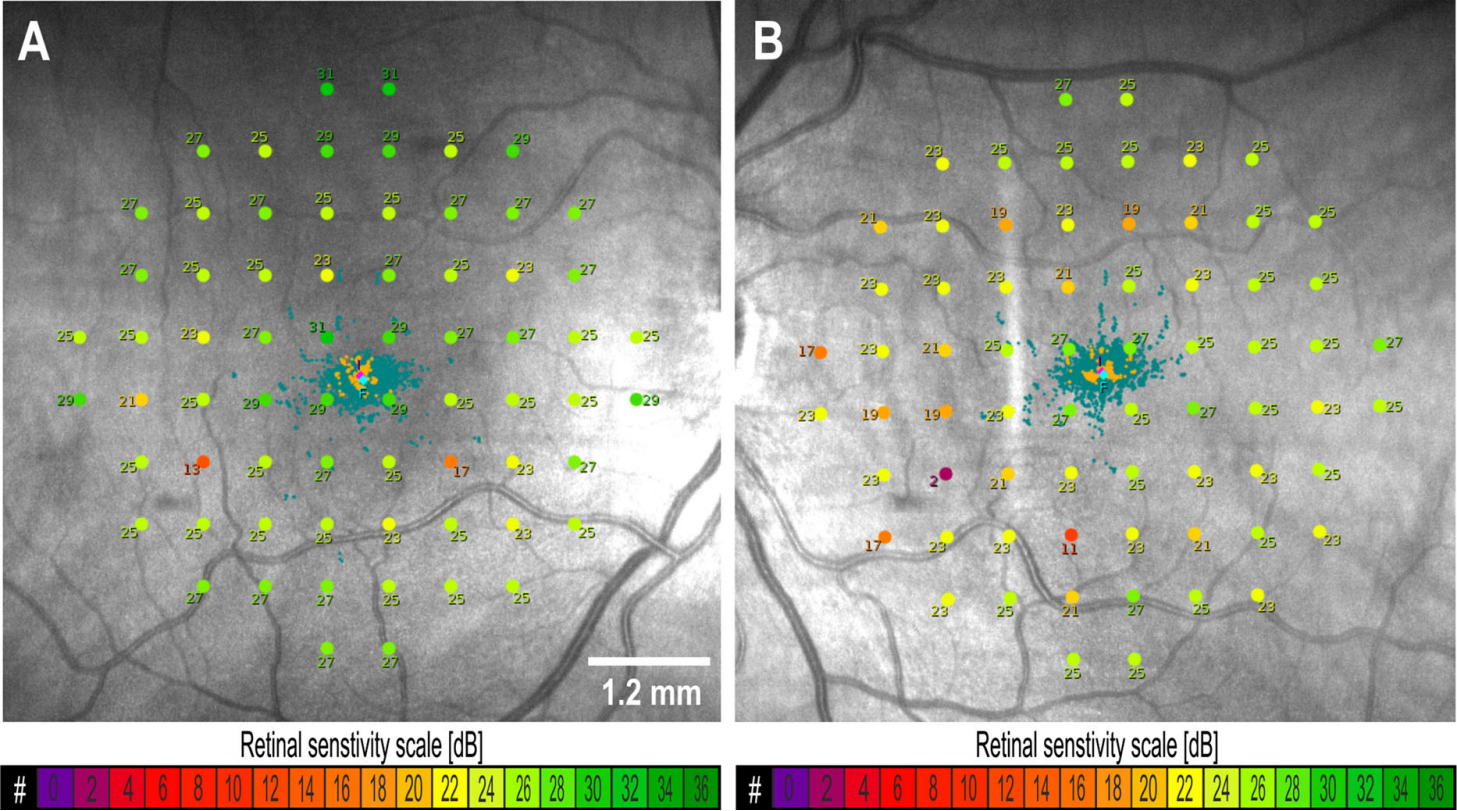
10–2 Macular Integrity Assessment (MAIA) microperimetry showing measured retinal sensitivity values in the right (**a**) and left (**b**) maculae in 2015. The location of fixation is shown by the dots scattered at the foveal center. Color scales for the symbols are shown below the image. Normal sensitivity is between 25 and 35 deciBel (dB)

Microperimetry demonstrated reduced overall mean sensitivity of 26.5 and 24.7 dB in the right and left eyes in 2014 declining to 25.8 and 23.0 dB, respectively, in 2015 (Fig. 1). The proportion of loci that had sensitivities below 2 SD from the mean of a control sample were 6% (3/68) and 19% (13/68) in the right and left eyes in 2014 increasing to 10% (7/68) and 37% (25/68), respectively, in 2015. Similar to Humphrey field test results, significant loss of sensitivity was noted at 3°, 5°, 7° and 9° eccentricities forming an incomplete paracentral ring (Supp Fig 2).

**Fig. 2:**
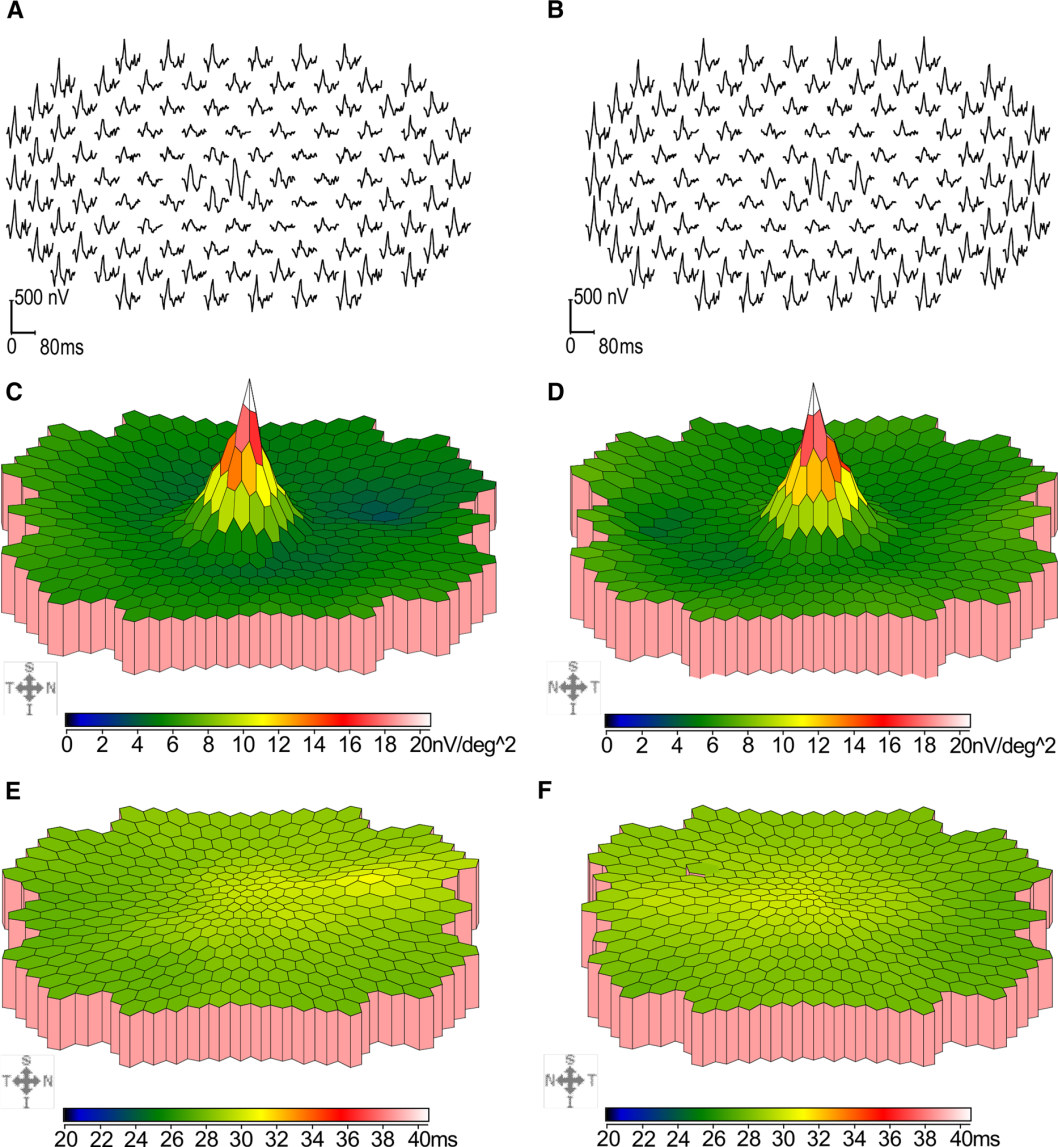
Multifocal electroretinography trace arrays of the right (**a**) and left (**b**) eye displayed in a retinal view perspective (S = superior, T = temporal, N = nasal and I = inferior) showing reduced amplitude densities in rings 3 and 4 in both eyes. Scalar plots for amplitude densities in right (**c**) and left (**d**) eyes and implicit times in right (**e**) and left (**f**) eyes are shown in nanoVolt (nV)/degree^2^ and millisecond (ms) scales (color bars)

The amplitude density of mfERG was relatively well preserved in the central hexagon (ring 1, covering the central 3°) and the adjacent hexagons (ring 2, annulus from 1.5° to 4° eccentricity). However, the density was significantly reduced in ring 3 (annulus from 4° to 8° eccentricity) in the right eye (Fig. 2). Both eyes had significantly reduced ring ratios at rings 3 and 4 (Supp Fig 3) which corresponds to an annulus from 4° to 12° eccentricity. The implicit times were increased in only some of the hexagons, but not as severe or extensive as the reduction in amplitude density when compared to controls (Supp Fig 3). Similar to both Humphrey field test and MAIA microperimetry, the functional defect on mfERG did not form a complete paracentral ring (Supp Fig 4).

### Structural assessment

**Fig. 3:**
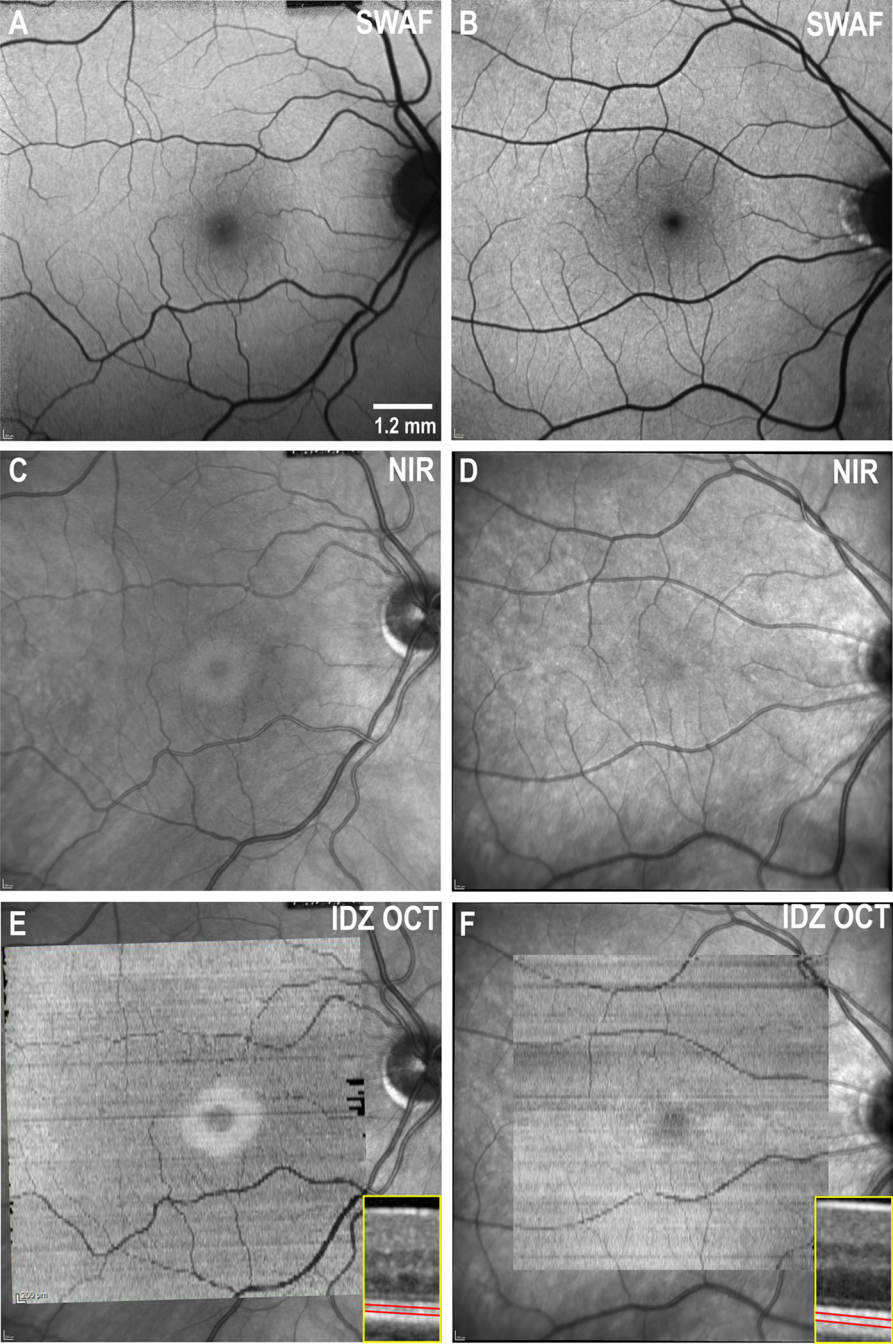
A 30° × 30° short-wavelength fundus autofluorescence (SWAF) imaging of the right eye in hydroxychloroquine (HCQ) toxicity case (**a**) and a control healthy subject (**b**) showing no obvious differences in autofluorescence signal from the foveal and perifoveal region. Near-infrared reflectance (NIR) imaging from the patient (**c**) shows an annular oval region of increased reflectance with an inner diameter of 410 µm and an outer diameter of 1450 µm horizontally and 430 and 1410 µm vertically. This bull’s eye ring lesion is absent in the healthy control (**d**). *En face* visualization of the interdigitation zone (IDZ) on optical coherence tomography (OCT), as shown in the insert (red lines demarcate the IDZ located between ellipsoid and retinal pigment epithelium), shows a region of preserved IDZ reflectivity coinciding with the NIR bull’s eye lesion in the case of HCQ toxicity (**e**), but there is no such ring in controls (**f**)

The 30° SWAF was within normal limits (Fig. 3a, b). However, the 30° NIR images revealed a subtle foveal ring of relative hyper-reflectivity resembling a bull’s eye lesion (Fig. 3c) which is not present in normal eyes (Fig. 3d). The outer radius of this ring was approximately 725 µm (2.42° of visual angle), and this boundary corresponded to attenuation of the interdigitation zone on OCT (Supp Fig 5) as illustrated in the *en face* OCT. In contrast, the ellipsoid zone was intact throughout the macular region. There was a prominent hyper-reflective disk in the *en face* reflectivity map of the interdigitation zone (Fig. 3e) compared to the relatively uniform reflectance in the healthy control eye (Fig. 3f). This hyper-reflective disk in the *en face* OCT (Fig. 3e) shows a striking resemblance to the bull’s eye lesion on NIR (Fig. 3c).

Retinal thicknesses and volumes were significantly reduced in the inner ring zones in both eyes (Supp Fig 6). There was a predilection for thinning in the temporal inner (500–1500 µm or 1.67°–5° eccentricity) and outer (1500–3000 µm or 5°–10° eccentricity) ring zones. Central subfield (central 1000 µm or 3.33°) thicknesses and volumes were within normal range.

**Fig. 4:**
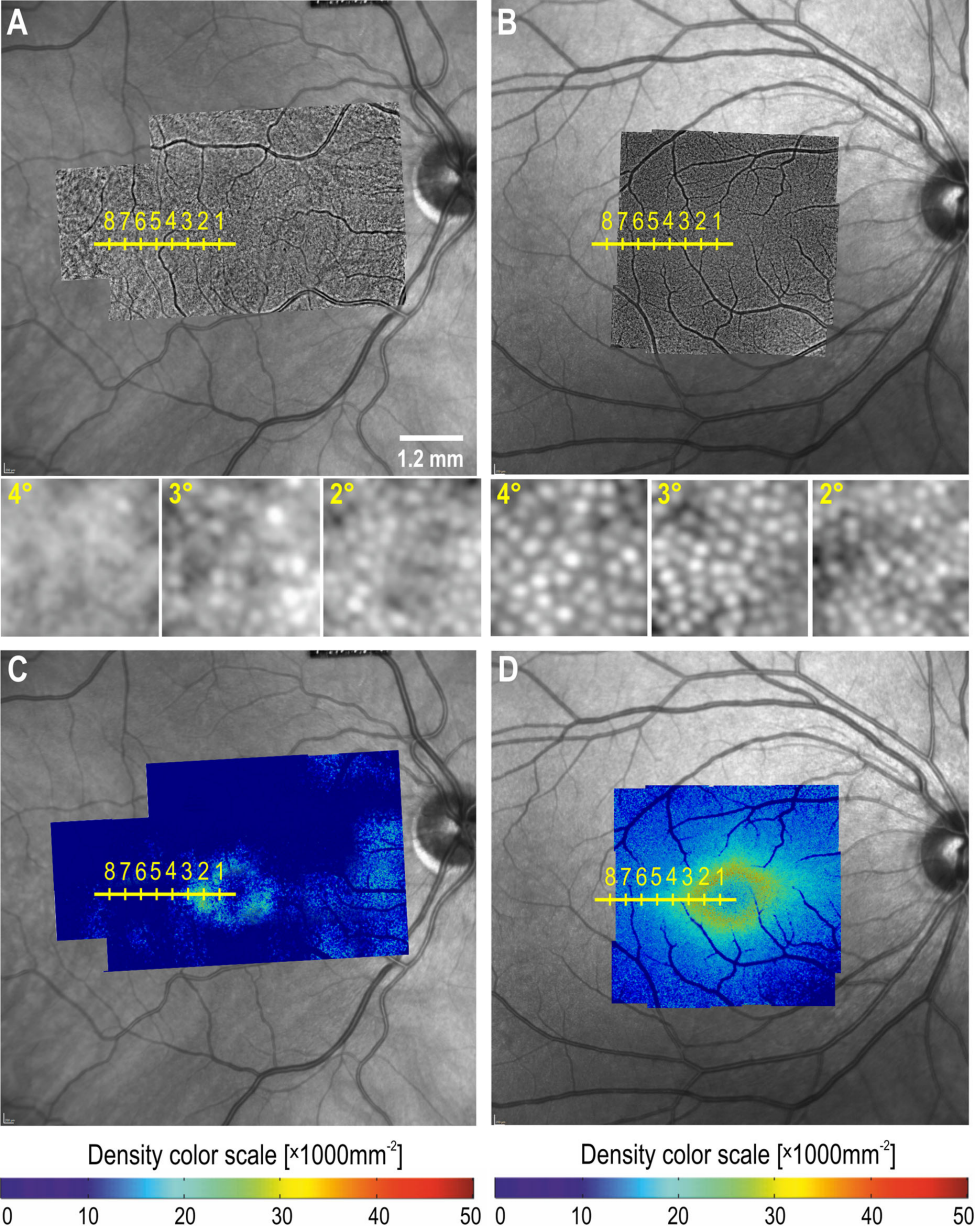
Overlay of the montage of adaptive optics (AO) cone images over near-infrared reflectance in the patient with toxicity (**a**) and a healthy subject (**b**) with a horizontal scale (yellow) marking the spacing between one degree from the foveal center. Insert shows zoomed-in images of cone reflexes at 2°, 3° and 4° eccentricities demonstrating the difference in cone packing at 3° and 4°, but not at 2°. Bright round signals represent wave-guiding cone outer segments. Overlay of cone density color map on near-infrared reflectance in the patient with toxicity (**c**) and a healthy subject (**d**) showing a central zone of reduced density due to inability of the rtx1 AO camera to resolve cones at the foveal center. Peak cone density is normally found at 2° eccentricity, and this is also seen in the eye with the bull’s eye lesion. However, the density of wave-guiding cones drops off rapidly from 3° eccentricity, outside the region of the bull’s eye lesion as seen on near-infrared reflectance. Color scale of cone density is shown below

**Fig. 5:**
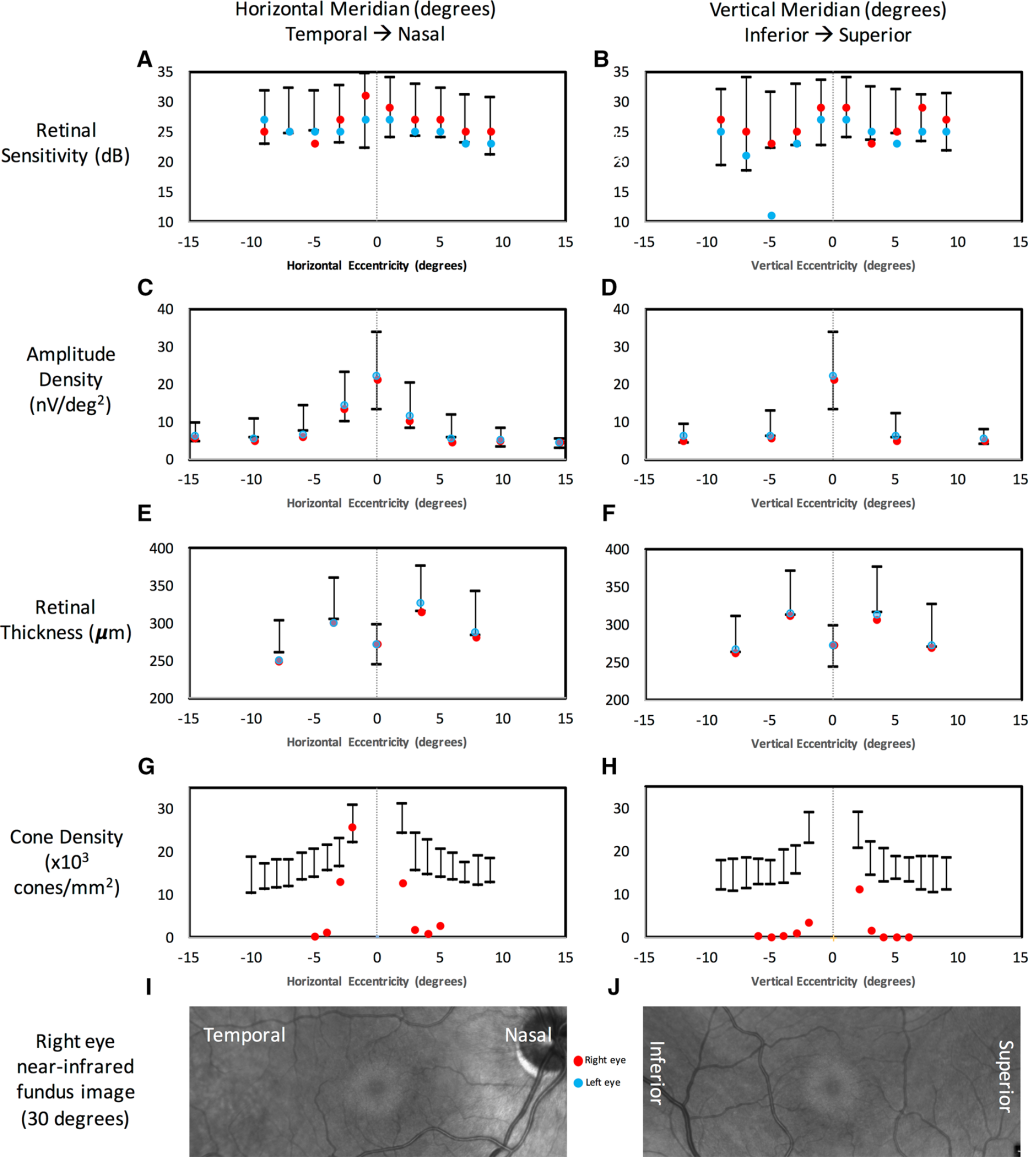
Graphical display of retinal sensitivity in deciBel (**a**, **b**), amplitude density of multifocal electroretinography (**c**, **d**), retinal thickness on optical coherence tomography (**e**, **f**) and cone density on adaptive optics retinal imaging (**g**, **h**) in the horizontal (left side) and vertical (right side) meridians. *X*-axis shows eccentricity in degrees from foveal center. Corresponding near-infrared reflectance images (**i**, **j**) showing the extent of the bull’s eye lesion. Bars represent −2 to +2 standard deviation of the normative data. Red circles = right eye data. Blue circles = left eye data. *X*-axis location of the measured value corresponds to exact location or the center of the zone of measurement. Retinal thickness is taken from central subfield, inner ring and outer ring of the Early Treatment for Diabetic Retinopathy Study grid

Densely packed cone outer segment signals were noted on wide-field AO montage within the boundary of the NIR bull’s eye lesion (Fig. 4). Cone density at 2° temporal eccentricity was 25,400 per mm^2^, within the expected normal interval (21,900–30,700 per mm^2^) derived from 19 healthy control eyes. At 3°, 4° and 5° eccentricity (beyond the boundary of the bull’s eye lesion), wave-guided signals were sparse and barely visible (12,900 and 1200 and 0 cones/mm^2^, respectively). A wide-field color map of the cone density (adjusted to axial length) shows reduced wave-guiding cone density within 2° of foveal center due to the inability of the AO device to resolve foveal cones. There was a significant reduction in wave-guiding cone density beyond 3° eccentricity compared to the control subject. The AO device derived cone density peaks at 2°–3° of retinal eccentricity in healthy subjects (Fig. 5).

### Structure–function correlation

To determine the functional impact of the observed structural changes, we plotted the retinal sensitivity, amplitude density, retinal thickness and cone density against eccentricity from the foveal center (Fig. 5). Retinal function was normal within the bull’s eye lesion while retinal sensitivity and amplitude density were reduced in some of the test locations in the region surrounding the bull’s eye lesion. The retinal dysfunction was non-uniform and did not form a symmetrical ring defect as shown in Humphrey field (Supp Fig 1), microperimetry (Supp Fig 2) and mfERG (Supp Fig 4). The corresponding reduction in retinal thickness and cone density outside 3° eccentricity was also asymmetrical (Supp Fig 6).

## Discussion

In summary, we present the multimodal imaging results of early HCQ toxicity to illustrate the anatomical and functional correlates of the subclinical NIR bull’s eye lesion. Our data suggest that the bull’s eye lesion is in fact not the region of pathology. Paradoxically, it is the area surrounding the bull’s eye lesion that was shown to have reduced NIR reflectance, interdigitation zone attenuation and loss of outer segment cone signals on AO imaging. This concept is supported by the subtle functional loss (microperimetry and mfERG) in the region immediately surrounding the bull’s eye.

The American Academy of Ophthalmology guidelines for ophthalmic screening of HCQ toxicity recommend a series of functional and structural investigations to screen and confirm HCQ maculopathy because bull’s eye maculopathy is a late clinical sign [7]. It has been recognized that 10% of patients with classic ring scotoma due to HCQ toxicity do not have obvious structural change on OCT [7]. However, a recently published work illustrated a very early sign of HCQ toxicity, a bull’s eye lesion on NIR imaging, before the loss of ellipsoid zone on OCT [1]. Jacob et al. [8] also showed that the hyper-reflective NIR bull’s eye lesion in HCQ toxicity corresponded to a region of intact interdigitation zone and normal cone structures up to, but not beyond 2° eccentricity. Our case illustrates the apparent structure–function mismatch as described by Marmor and Melles [7]. However, detailed image analysis using *en face* reconstruction of the interdigitation zone highlighted abnormalities that are not easily appreciated on cross-sectional OCT images. The interdigitation zone reflectivity map mirrors the NIR image and hence confirms our hypothesis that the hyper-reflective bull’s eye lesion represents relative sparing of the cone photoreceptor outer segment rather than a region of RPE abnormality as suggested by Wong et al. [1]. Recent AO imaging and OCT studies have also shown that interdigitation zone integrity correlates with reflectance of cone outer segments [8]. Hence, the mechanism of hyper-reflective NIR bull’s eye lesion can be explained by the loss of perifoveal interdigitation zone (outer segment tips) through HCQ toxicity.

We also conducted extensive structure–function analysis to determine the functional impact of this early toxicity sign. Our hypothesis of preserved interdigitation zone within the bull’s eye lesion is further supported by the normal retinal sensitivity (microperimetry) and amplitude density (mfERG) within the region of the hyper-reflective ring. Conversely, there was a reduction in retinal sensitivity from 3° to 9° eccentricity and a reduction in amplitude density within hexagons from rings 3 to 4 of the mfERG array (4°–12° eccentricity). Although the retinal dysfunction in this case was not radially symmetrical (i.e., partial ring defects), reduction in ring ratios demonstrated the typical distribution of HCQ toxic effect. It is unknown why there is a discordance between the uniform attenuation in interdigitation zone surrounding the bull’s eye and the patchy asymmetrical thinning of the perifoveal retina, retinal sensitivity loss and mfERG abnormalities. Nevertheless, our functional data are also consistent with Wong et al.’s [1] observation of a case of advanced toxicity where central field defect was accompanied by loss of the foveal hyper-reflective ring on NIR imaging. This can be explained by progressive reduction in the size of the central hyper-reflective ring over time as the photoreceptor outer segments are progressively lost in a centripetal fashion in the late stages of HCQ toxicity. Thus, we anticipate that if our patient had continued to take HCQ, the interdigitation zone within the bull’s eye lesion would eventually be lost (with disappearance of the bull’s eye lesion) and field defect would encroach into central test loci of the 10–2 grid on microperimetry and reduced mfERG ring ratio would also involve ring 2 of the mfERG array.

Ring lesions in the fovea have also been seen in rod–cone dystrophy where a preserved foveal island of cone photoreceptors is surrounded by a bright ring of hyper-autofluorescence on short-wavelength excitation due to loss of photoreceptor outer segments [9]. However, the OCT signs that contribute to the NIR bull’s eye lesion are much more subtle in HCQ maculopathy than in rod–cone dystrophy because the loss is restricted to the interdigitation zone in HCQ toxicity rather than the entire outer retina in retinal dystrophy. Hence, the reconstruction of interdigitation zone *en face* OCT may complement NIR imaging in the monitoring of HCQ toxicity, especially in the 10% of patients in whom ring scotoma developed before ellipsoid zone loss on OCT [7]. The absence of RPE abnormality on OCT and short-wavelength autofluorescence is consistent with the notion that visible RPE damage is not an early clinical feature of HCQ toxicity [10].

Although this is the first report illustrating the structural basis of the NIR bull’s eye lesion and its functional consequences, there are several limitations. First, visual field assessment showed only incomplete ring scotoma with radial asymmetry, but the use of ring ratios in assessing mfERG amplitude densities and implicit times provided objective evidence of ring-like functional deficit. This underscores the importance of using an objective functional investigation to confirm results of more variable subjective tests such as Humphrey perimetry or MAIA microperimetry in HCQ toxicity. Second, flood-illumination AO retinal imaging is unable to resolve rod or foveal cones outer segments, and therefore, we were therefore unable to exclude rod defects or central cone loss within the NIR bull’s eye lesion. Another limitation of the flood-illumination AO system is the inability to visualize cones with damaged outer segment tips that are unable to reflect the wave-guided signal. This results in underestimation of cone density in the regions where outer segments might be preferentially affected by toxicity. The use of scanning laser ophthalmoscope-based system can overcome these obstacles because of its higher resolution in confocal mode and the ability to visualize cone inner segment in non-confocal split detection mode [11]. Third, there is a wide interindividual variation in cone density across the macular region which reduces the ability of AO imaging in detecting subtle reduction in cone density [12, 13]. Fourth, it is important to note that images obtained using the AO retinal camera can vary significantly in quality. This is particularly true for those images obtained using flood-illumination AO devices where even in young healthy subjects with no apparent eye disease, the quality of the images can be so poor that automated programs for quantifying cone density are not able to compute the desired measurements [14]. Even where images of adequate quality are obtained, cone density calculations can be highly variable both within and between different methods of cone counting [15]. Additionally, the entry pupil may alter the appearance of cones and hence the calculation of cone density. This has been shown to be the case in the retinal periphery by Miloudi et al. [16], but the authors commented that this effect was not observed in the macula. Fifth, the construction of *en face* OCT reflectivity maps has recently been described in the visualization of subretinal drusenoid deposits [17]. However, the methodology of *en face* OCT has not been standardized. We have created interdigitation zone *en face* OCT map in many healthy eyes and have not seen any ring-like structures as demonstrated in the case of HCQ toxicity. Sixth, we used a relatively small sample of healthy subjects to derive normative range and calculate standard deviation. However, the use of modified *t* test eliminates the higher rate of type I error when small control sample is used to derive a *z*-score; hence, we are less likely to erroneously classify results as abnormal when in fact it is normal [5].

In conclusion, multimodal imaging demonstrated that the NIR bull’s eye lesion is in fact not the site of pathology. The bull’s eye seen on NIR comes from reduced reflectance, interdigitation zone attenuation and reduced wave-guiding cone density in the surrounding region. These structural changes co-localized with region of functional loss, in the form of a ring-like scotoma, and mfERG abnormalities. Further studies are required to investigate whether the bull’s eye on NIR imaging and *en face* OCT is prominent or consistent enough for diagnostic use.

## Electronic supplementary material

Below is the link to the electronic supplementary material.

Supp Fig. 1 10–2 Humphrey automated perimetry output in a field view perspective for the left (A) and right (B) eyes retinal threshold values and deviation from the mean of a healthy population. The symbols in the total and pattern deviation plots denote retinal threshold deviations that were in the bottom 5, 2 or 1 percentile of healthy population based on non-Gaussian statistics provided by the Humphrey field perimeter. (TIFF 341 kb)

Supp Fig. 2 10–2 Macular Integrity Assessment (MAIA) microperimetry presented in a normalized deviation plot in retinal view perspective showing locations where measurement values were -2.00 to -2.99 standard deviations (yellow) or -3.00 or more standard deviations (red) from the expected mean derived from 36 healthy controls. Results from the right (A) and left (B) eyes in 2014 and right (C) and left (D) eyes in 2015 showed worsening of pericentral incomplete ring scotoma with increased number of loci having sensitivity dropping below -2 or -3 standard deviations from normal. Note that the central 4 loci at 1° and 1° eccentricity are spared except in the left eye in 2014. (TIFF 899 kb)

Supp Fig. 3 Multifocal electroretinography presented in a normalized deviation plot in retinal view perspective showing a ring-like zone of amplitude density reduction (toward dark green color: negative standard deviation) in the right (A) and left (B) eyes and corresponding region of implicit time delay (toward orange-red color: positive standard deviation) in the right (C) and left (D) eyes. Ring ratio (Rx:R6) plot showing significant reduction in response density at 4° to 12° eccentricity (red colored region) in right (E) and left (F) eyes, but the implicit time delay did not exceed the expected normal range (shown in green). *Y*-axis is the ring ratio, and *x*-axis is the retinal eccentricity from fixation in degrees. Red line denotes normal mean. Black line denotes patient data. Green zone denotes normative range within 2 standard deviations from the mean. Red zone denotes deviation of patient line outside the normative range. The smoothed ring ratio plot is a cubic spline interpolation provided by the manufacturer to facilitate visualization. (TIFF 1798 kb)

Supp Fig. 4 Multifocal electroretinography presented in a normalized deviation plot in a retinal view perspective showing stimulus locations where measurement values were 2.00 to 2.99 standard deviation (yellow) or 3.00 or more standard deviations (red) from the expected mean derived from a control cohort. A complete ring of reduced amplitude density is seen in the right (A), but only a partial ring is present in the left (B) eye. Implicit time delay is only found in scattered locations forming an incomplete ring in both the right (C) and left (D) eyes. Note that rings 1 (central hexagon) and 2 (6 surrounding hexagon) have response densities and implicit times within 2 standard deviation of mean from the control sample. (TIFF 9938 kb)

Supp Fig. 5 Near-infrared reflectance (NIR) image (A) and optical coherence tomography (OCT) image (B) of the patient with hydroxychloroquine (HCQ) toxicity showing a bull’s eye lesion and its correlation with interdigitation zone attenuation between 2° and 3° of eccentricity. Yellow scale marks visual angle in degrees of eccentricity, and the inserts below the horizontal OCT scan show zoomed images of the four outer hyper-reflective bands corresponding to external limiting membrane, ellipsoid zone, interdigitation zone and retinal pigment epithelium, respectively, at 0°, 1, 2°, 3°, 4° and 5° of retinal eccentricities. In the healthy control eye, NIR (C) shows no bull’s eye lesion and OCT (D) shows no attenuation of the interdigitation zone up to 5° of eccentricity (insert denoting retinal location in visual angle). (TIFF 1939 kb)

Supp Fig. 6 Retinal thickness map in the Early Treatment of Diabetic Retinopathy Study grid presented in a normalized deviation plot in a retinal view perspective showing regions where thickness (A, B) and volume (C, D) were -2.00 to -2.99 standard deviation (yellow) from the expected mean derived from a control cohort. In all eyes, the central 1-mm zone thickness and volume were within normal range. The right (A) eye retinal thickness map shows retinal thinning of all quadrants in the inner ring (diameter 1 to 3 mm) and only the temporal zone of the outer ring (diameter 3 to 6 mm). The left eye (B) retinal thickness map shows thinning in the temporal and superior zones of the inner ring and temporal zone of the outer ring. There is also reduction in retinal volume in temporal, superior and inferior zones of the inner ring and temporal zones of the outer ring in right (C) and left (D) eyes. (TIFF 4076 kb)
