# Supplementary figures and images for: Perifoveal interdigitation zone loss in hydroxychloroquine toxicity leads to subclinical bull’s eye lesion appearance on near-infrared reflectance imaging
Source: Doc Ophthalmol. 2017 Nov 9;136(1):57–68. doi: 10.1007/s10633-017-9615-9 (PMC5811575; doi:10.1007/s10633-017-9615-9)

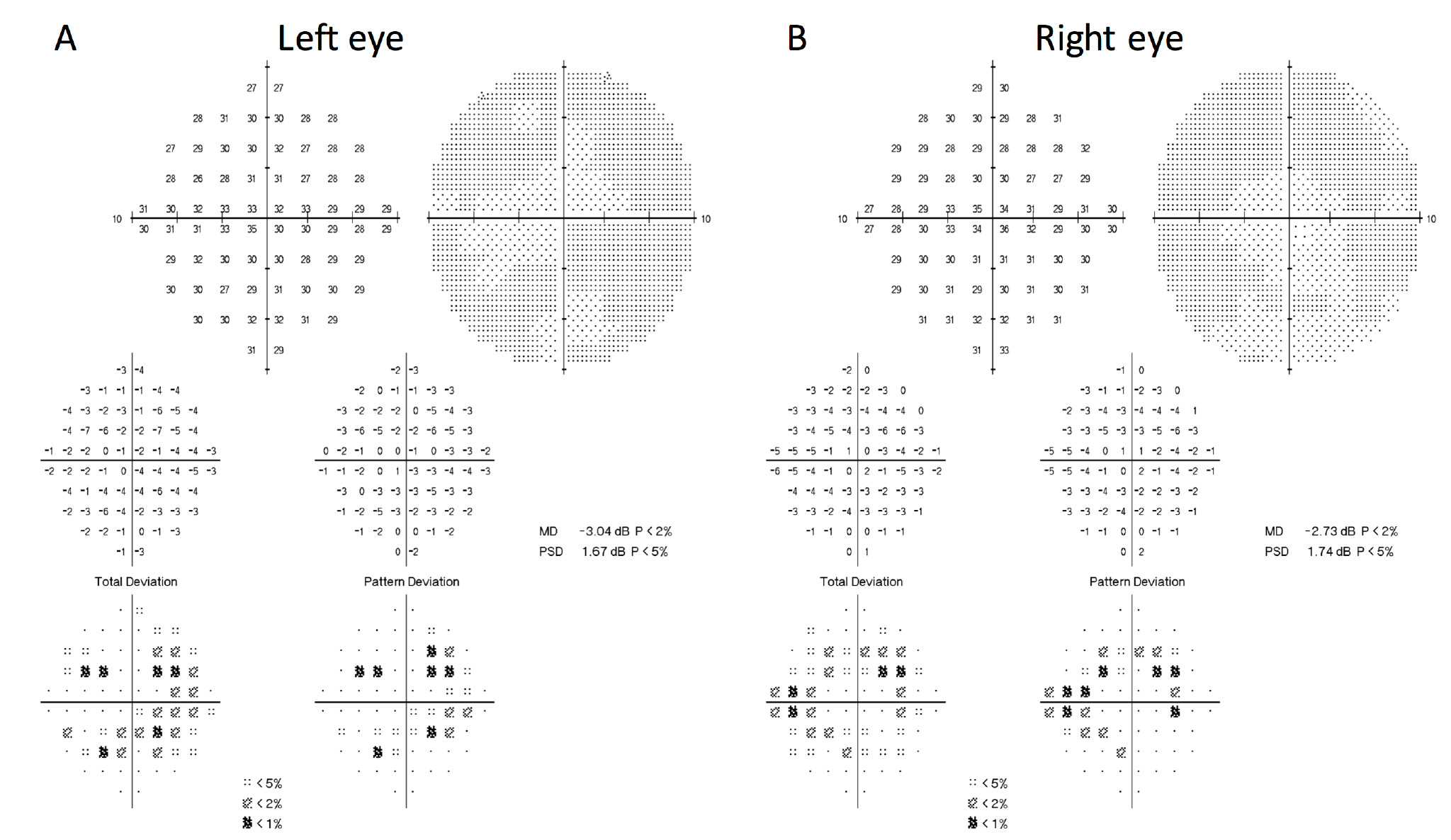

Supplement: Supplementary file 1 — 10–2 Humphrey automated perimetry output in a field view perspective for the left (A) and right (B) eyes retinal threshold values and deviation from the mean of a healthy population. The symbols in the total and pattern deviation plots denote retinal threshold deviations that were in the bottom 5, 2 or 1 percentile of healthy population based on non-Gaussian statistics provided by the Humphrey field perimeter. (TIFF 341 kb) [file 10633_2017_9615_MOESM1_ESM.tiff]

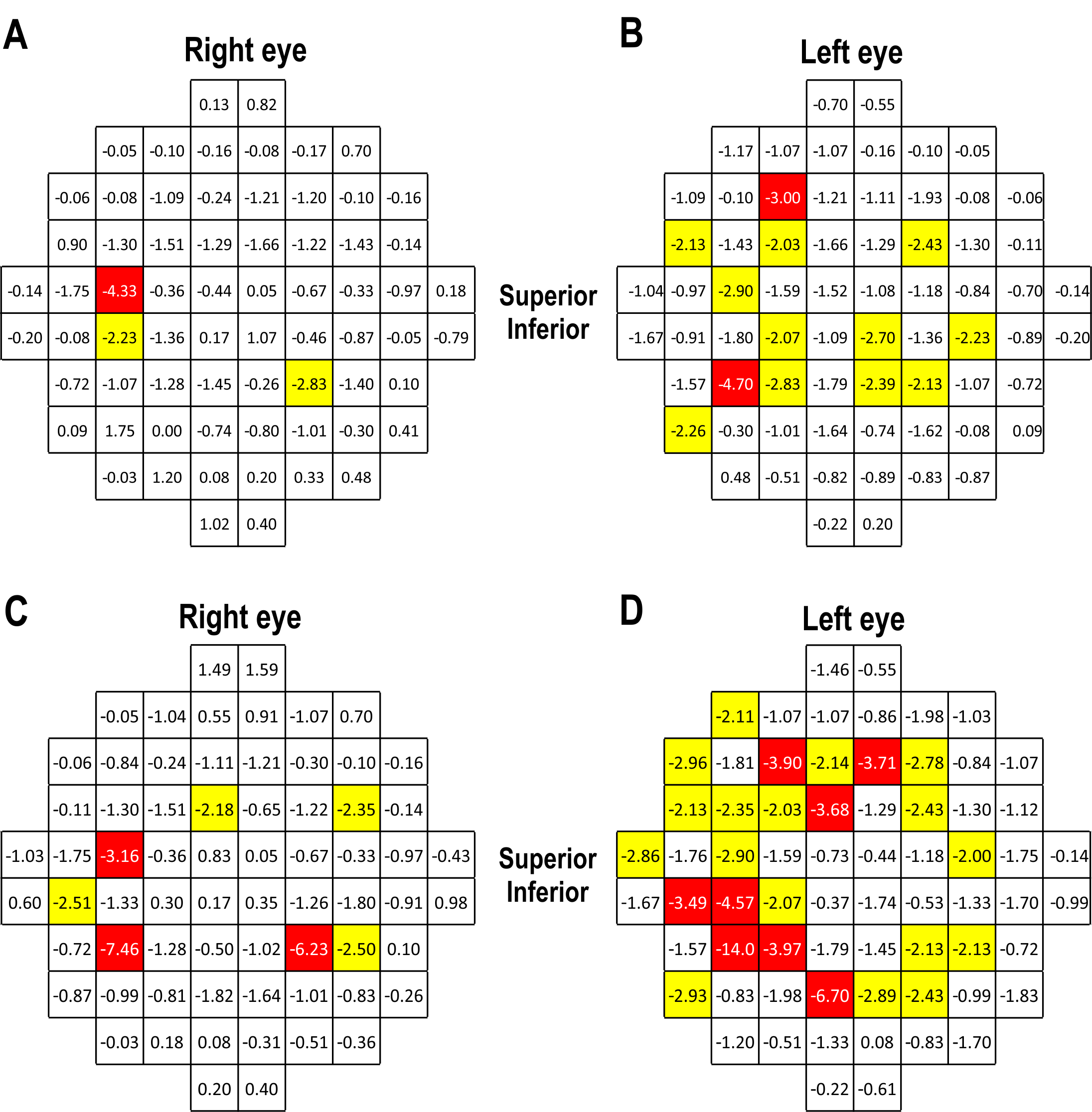

Supplement: Supplementary file 2 — 10–2 Macular Integrity Assessment (MAIA) microperimetry presented in a normalized deviation plot in retinal view perspective showing locations where measurement values were -2.00 to -2.99 standard deviations (yellow) or -3.00 or more standard deviations (red) from the expected mean derived from 36 healthy controls. Results from the right (A) and left (B) eyes in 2014 and right (C) and left (D) eyes in 2015 showed worsening of pericentral incomplete ring scotoma with increased number of loci having sensitivity dropping below -2 or -3 standard deviations from normal. Note that the central 4 loci at 1° and 1° eccentricity are spared except in the left eye in 2014. (TIFF 899 kb) [file 10633_2017_9615_MOESM2_ESM.tif]

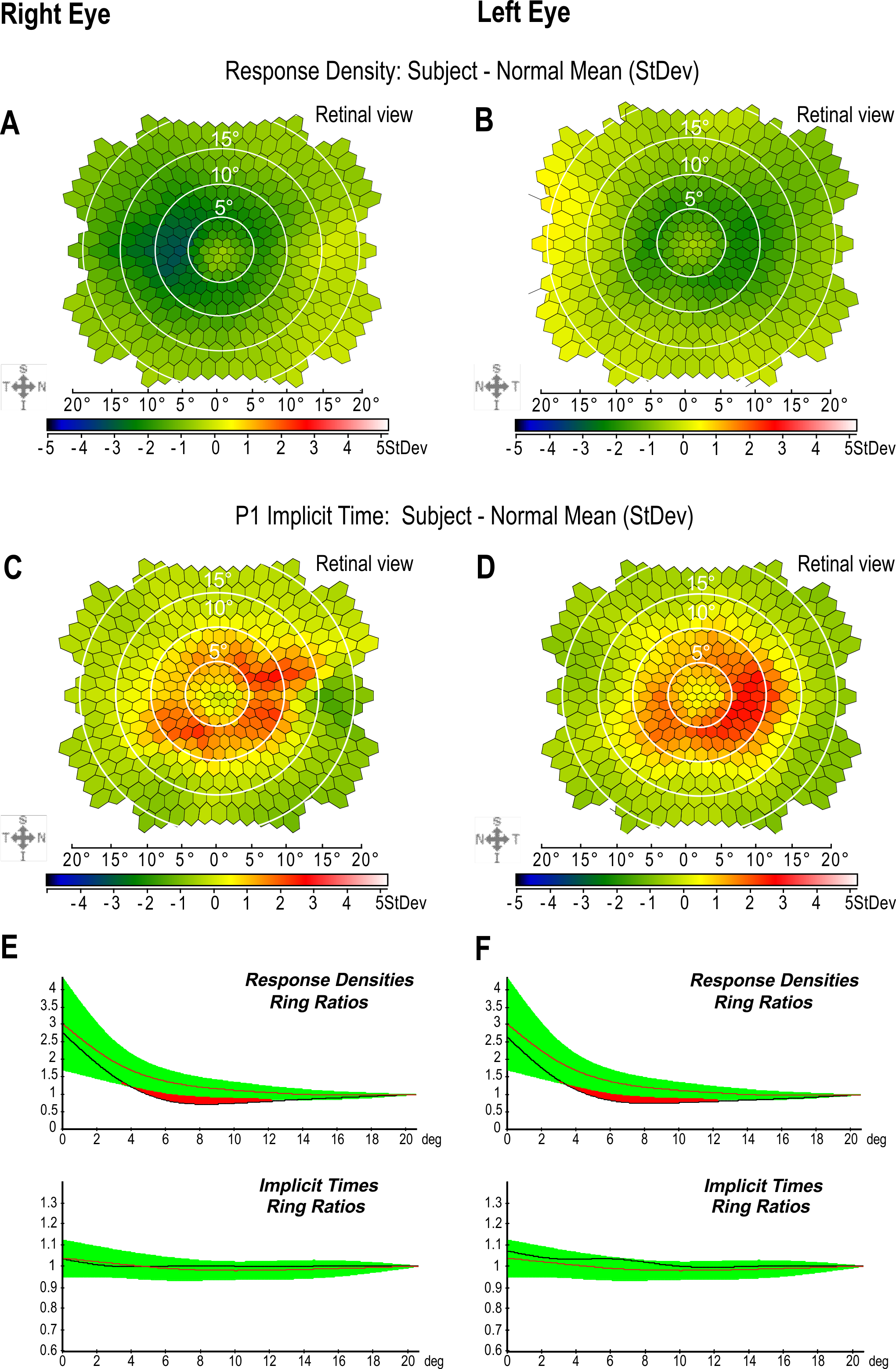

Supplement: Supplementary file 3 — Multifocal electroretinography presented in a normalized deviation plot in retinal view perspective showing a ring-like zone of amplitude density reduction (toward dark green color: negative standard deviation) in the right (A) and left (B) eyes and corresponding region of implicit time delay (toward orange-red color: positive standard deviation) in the right (C) and left (D) eyes. Ring ratio (Rx:R6) plot showing significant reduction in response density at 4° to 12° eccentricity (red colored region) in right (E) and left (F) eyes, but the implicit time delay did not exceed the expected normal range (shown in green). Y-axis is the ring ratio, and x-axis is the retinal eccentricity from fixation in degrees. Red line denotes normal mean. Black line denotes patient data. Green zone denotes normative range within 2 standard deviations from the mean. Red zone denotes deviation of patient line outside the normative range. The smoothed ring ratio plot is a cubic spline interpolation provided by the manufacturer to facilitate visualization. (TIFF 1798 kb) [file 10633_2017_9615_MOESM3_ESM.tif]

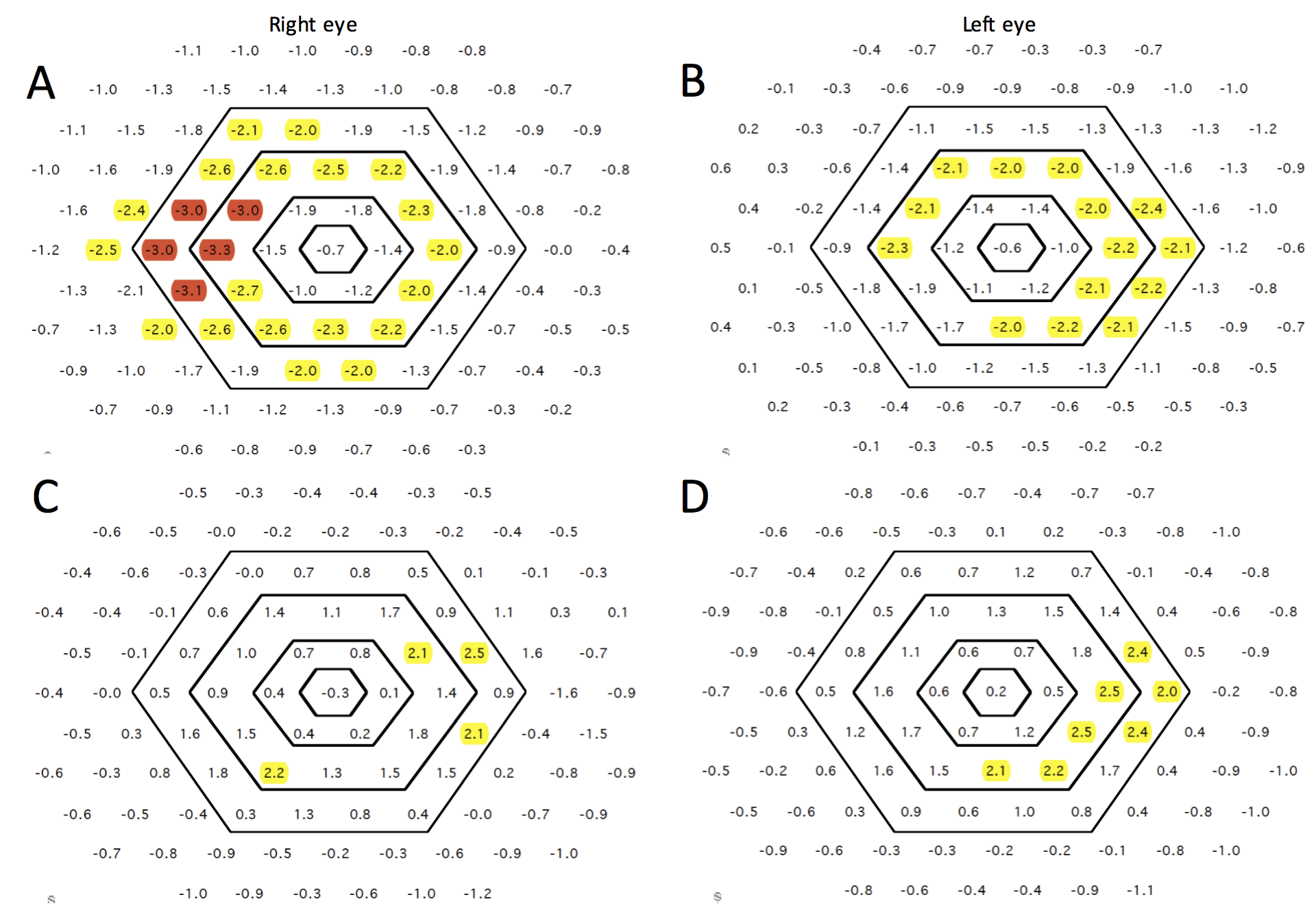

Supplement: Supplementary file 4 — Multifocal electroretinography presented in a normalized deviation plot in a retinal view perspective showing stimulus locations where measurement values were 2.00 to 2.99 standard deviation (yellow) or 3.00 or more standard deviations (red) from the expected mean derived from a control cohort. A complete ring of reduced amplitude density is seen in the right (A), but only a partial ring is present in the left (B) eye. Implicit time delay is only found in scattered locations forming an incomplete ring in both the right (C) and left (D) eyes. Note that rings 1 (central hexagon) and 2 (6 surrounding hexagon) have response densities and implicit times within 2 standard deviation of mean from the control sample. (TIFF 9938 kb) [file 10633_2017_9615_MOESM4_ESM.tiff]

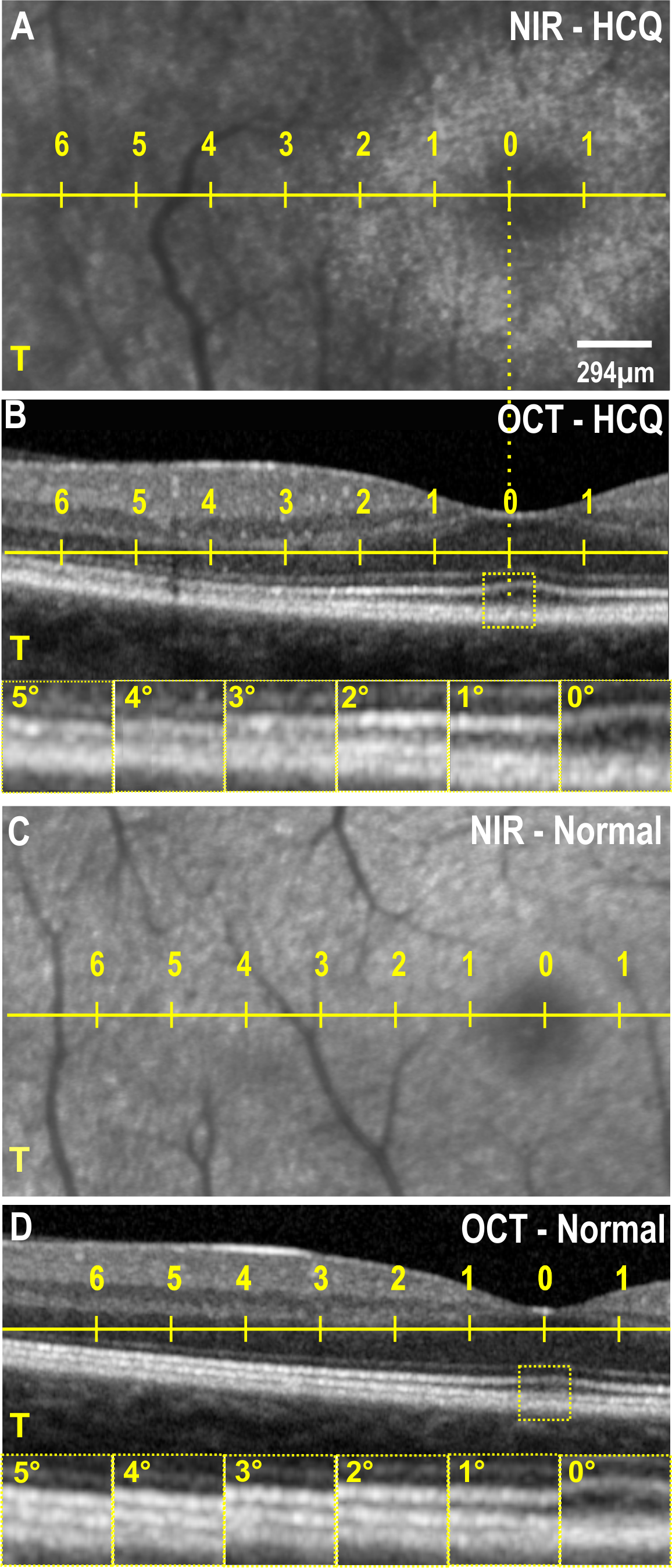

Supplement: Supplementary file 5 — Near-infrared reflectance (NIR) image (A) and optical coherence tomography (OCT) image (B) of the patient with hydroxychloroquine (HCQ) toxicity showing a bull’s eye lesion and its correlation with interdigitation zone attenuation between 2° and 3° of eccentricity. Yellow scale marks visual angle in degrees of eccentricity, and the inserts below the horizontal OCT scan show zoomed images of the four outer hyper-reflective bands corresponding to external limiting membrane, ellipsoid zone, interdigitation zone and retinal pigment epithelium, respectively, at 0°, 1, 2°, 3°, 4° and 5° of retinal eccentricities. In the healthy control eye, NIR (C) shows no bull’s eye lesion and OCT (D) shows no attenuation of the interdigitation zone up to 5° of eccentricity (insert denoting retinal location in visual angle). (TIFF 1939 kb) [file 10633_2017_9615_MOESM5_ESM.tif]
